# Prognostic performance of the FACED score and bronchiectasis severity index in bronchiectasis: a systematic review and meta-analysis

**DOI:** 10.1042/BSR20194514

**Published:** 2020-10-30

**Authors:** Min He, Min Zhu, Chengdi Wang, Zuohong Wu, Xiaofeng Xiong, Hongxia Wu, Deyun Cheng, Yulin Ji

**Affiliations:** 1 Health Management Center, West China Hospital of Sichuan University, Chengdu 610041, China; 2 Department of Respiratory and Critical Care Medicine, West China Hospital of Sichuan University, Chengdu 610041, China

**Keywords:** All-cause mortality, Bronchiectasis Severity Index, Bronchiectasis, FACED, Hospitalization, Severity score

## Abstract

**Background:** Bronchiectasis is a multidimensional lung disease characterized by bronchial dilation, chronic inflammation, and infection. The FACED (Forced expiratory volume in 1 s (FEV1), Age, Chronic colonization, Extension, and Dyspnea) score and Bronchiectasis Severity Index (BSI) are used to stratify disease risk and guide clinical practice. This meta-analysis aimed to quantify the accuracy of these two systems for predicting bronchiectasis outcomes.

**Methods:** PubMed, Embase, and the Cochrane Database of Systematic Reviews were searched for relevant studies. Quality of included studies was assessed using the Quality Assessment of Diagnostic Accuracy Studies-2 (QUADAS-2) criteria. Pooled summary estimates, including sensitivity, specificity, positive likelihood ratio (PLR), negative likelihood ratio (NLR), and diagnostic odds ratio (DOR) were calculated. Summary receiver operating characteristic curves were constructed, and the area under the curve (AUC) was used to evaluate prognostic performance.

**Results:** We analyzed 17 unique cohorts (6525 participants) from ten studies. FACED scores with a cut-off value ≥ 5 predicted all-cause mortality better than BSI with a cut-off value ≥ 9, based on pooled sensitivity (0.34 vs 0.7), specificity (0.94 vs 0.66), PLR (4.76 vs 2.05), NLR (0.74 vs 0.48), DOR (6.67 vs 5.01), and AUC (0.87 vs 0.75). Both FACED scores with a cut-off value ≥ 5 (AUC = 0.82) and BSI scores with a cut-off value ≥ 5 or 9 (both AUC = 0.80) help to predict hospitalization.

**Conclusions:** At a cut-off value ≥ 5, FACED scores can reliably predict all-cause mortality and hospitalization, while BSI scores can reliably predict hospitalization with a cut-off of ≥5 or ≥9. Further studies are essential to validate the prognostic performance of these two scores.

## Introduction

Bronchiectasis is a chronic inflammatory and structural lung disease characterized by chronic dilation of the bronchi; clinical symptoms of the disease include persistent cough, sputum production, and recurrent respiratory infections [1,2]. In recent years, bronchiectasis has become a major health concern due to its increasing prevalence and associated healthcare costs [1–4].

Due to the lack of effective treatment options, the current management strategies for bronchiectasis focus on controlling symptoms, reducing risk, avoiding exacerbation, and slowing disease progression [1,2]. Most recommended first-line treatments for bronchiectasis are long-term antimicrobial therapies, which are costly and can cause adverse events [1,2]. Thus, the first step in clinical decision-making should be stratifying bronchiectasis patients by risk of poor prognosis in order to target treatments to those most likely to experience a net benefit. Furthermore, clearly defined stratification of bronchiectasis patients would improve the comparability of populations analyzed in different research studies [5].

Three multidimensional severity scoring systems have been derived and validated for bronchiectasis: FACED, so named because it takes into account Forced expiratory volume in 1 s (FEV1), Age, Chronic colonization, Extension, and Dyspnea; EFACED (with Exacerbation added to FACED); and the Bronchiectasis Severity Index (BSI), which involves a 9-item scale encompassing demographic and clinical characteristics, as well as microbiological and radiological data [6,7]. FACED and BSI scores have been more extensively researched and are more widely used [6,7]. The FACED score is an easy-to-use grading system with an excellent predictive performance regarding mortality [7,8]. The BSI was developed and validated in a large multicenter study in Europe [9]. These prognostic scoring systems can help to predict mortality, hospitalization, and disease exacerbation, as well as to evaluate quality of life in patients with bronchiectasis. Although some studies have compared the accuracy of these scoring systems [10,11], whether one is better is unclear. This is an important question to address because some medical centers may lack the clinical experience or equipment to implement all scoring systems well.

Considerable effort needs to be invested to validate the prognostic performance of these scoring systems in varied settings before they can be accepted and extensively applied in research and clinical decision-making. In this meta-analysis, we aimed to quantify and compare the accuracy of the FACED and BSI systems at predicting disease outcomes (all-cause mortality, respiratory-related mortality, or hospitalization) in bronchiectasis patients. As part of the present study, we aimed to determine optimal cut-off values for each system as a basis for standardizing prognostic prediction.

## Methods

This systematic review and meta-analysis was registered with the PROSPERO database of systematic reviews (CRD42018096462).

### Search strategy

Two reviewers (M.H. and M.Z.) independently searched PubMed, Embase, and the Cochrane Database of Systematic Reviews to identify studies on bronchiectasis published before June 2019. The search strings are provided in the Supplementary material (e-Appendix 1). The reference lists in the included studies and in relevant review articles were screened manually.

### Study eligibility

After removing duplicate references, two reviewers (M.H. and M.Z.) independently screened the titles and abstracts of the potentially relevant studies, followed by a complete review of each relevant full text. Disagreements were resolved through discussion or consultation with the third author (C.W.). All included studies were written in English or Chinese, with no restrictions on study design. All included studies used the FACED system and/or BSI system to assess bronchiectasis (diagnosed using high-resolution chest computed tomography) and had sufficient data to directly or indirectly assess the predictive performance of the FACED system and/or BSI system regarding at least one outcome of interest (mortality and/or hospitalization).

If a study contained data from several cohorts, each cohort was treated as a separate study, consistent with established practices [12]. If research subjects and reported outcomes overlapped between studies, we combined the data from those studies and analyzed the data based on methods described in a previous study [13].

### Data extraction and quality assessment

Two reviewers (M.H. and M.Z.) independently assessed the quality of the included studies using the Quality Assessment of Diagnostic Accuracy Studies-2 (QUADAS-2) criteria [14]. Relevant data were extracted from the included studies using a standardized extraction form.

### Statistical analysis

Our analyses focused on the ability of the two scoring systems to predict all-cause mortality, respiratory-related mortality, or hospitalization, and on the agreement between the two scoring systems. Heterogeneity between studies was assessed and quantified using the *I^2^* statistic. Based on the heterogeneity observed, random-effects (*I^2^* > 50%) or fixed-effects (*I^2^* ≤ 50%) models were used to calculate summary estimates, including sensitivity, specificity, positive likelihood ratio (PLR), negative likelihood ratio (NLR), and diagnostic odds ratio (DOR). We generated summary receiver operating characteristic curves (SROCs) and calculated the area under the SROC (AUC) values. Meta-regression was performed to explore the sources of statistically significant heterogeneity, followed by subgroup analyses. Publication bias was assessed using Deeks’ test. Consensus between the scoring systems was evaluated using the kappa (κ) coefficient and chi-squared test. Statistical analysis was performed using RevMan 5.3 (Cochrane Collaboration), Meta-DiSc 1.4 (XI Cochrane Colloquium, Barcelona, Spain), and Stata 12.0 (Stata, College Station, TX, U.S.A.).

## Results

### Literature screening and assessment

**Figure 1:**
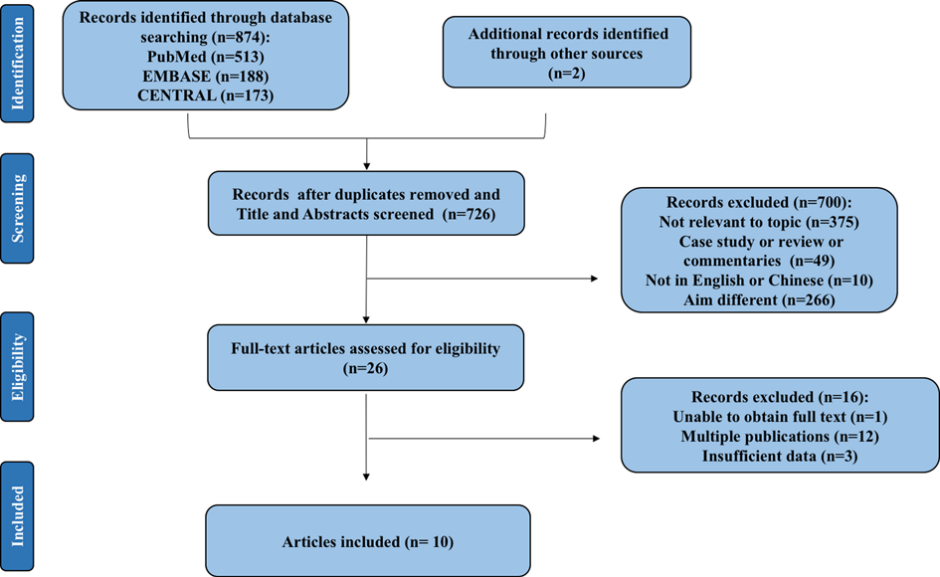
Flowchart of study selection

**Figure 2:**
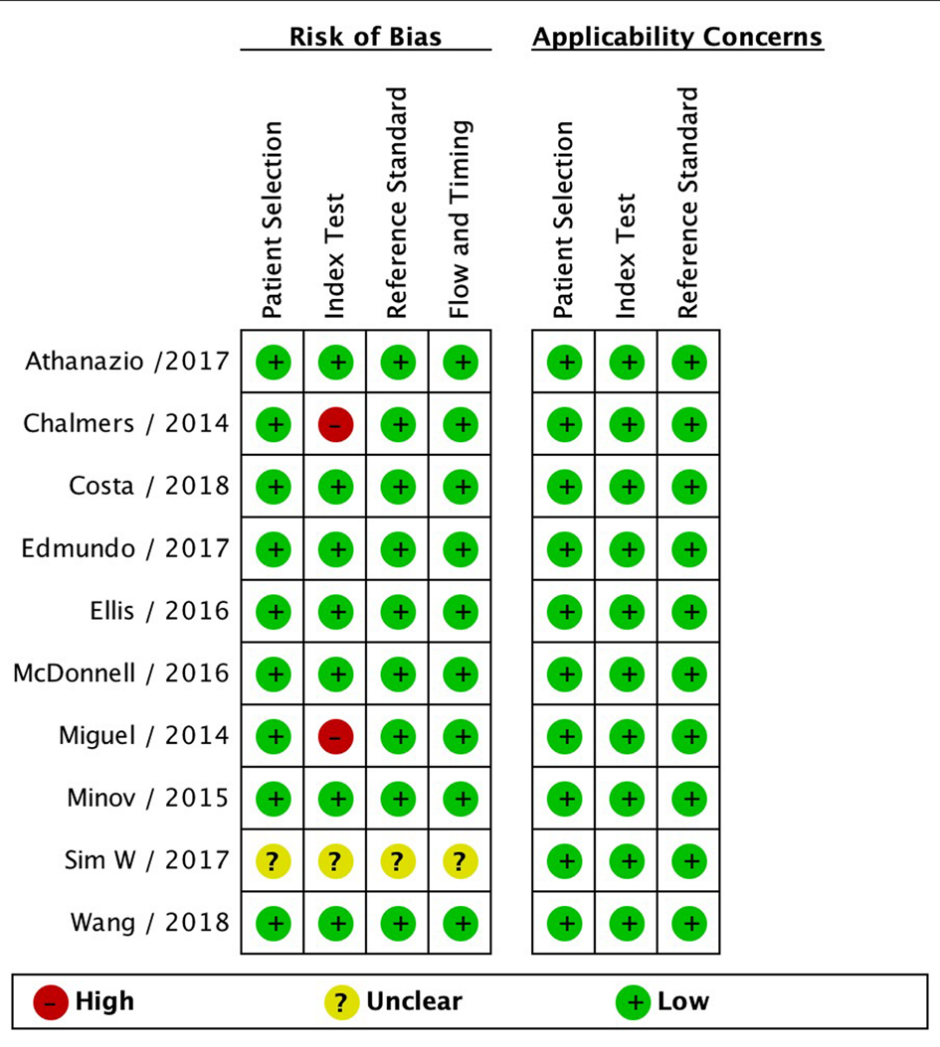
Quality assessment of included studies using the QUADAS-2 criteria

After a detailed assessment based on the eligibility criteria, the final meta-analysis included 17 unique cohorts with 6525 participants across ten publications (Figure 1) [8–11,15–20]. The characteristics of the included studies are summarized in Table 1. The QUADAS-2 assessment demonstrated that most of the included studies had a low risk of bias, indicating the reliability of the statistical results (Figure 2). For the initial analysis, we stratified the bronchiectasis patients into three groups: mild (BSI score, 0–4; FACED score, 0–2), moderate (BSI, 5–8; FACED, 3–4), or severe (BSI, 9; FACED, 5–7). Patients in these three groups were compared in terms of age, rates of all-cause or respiratory-related mortality, and rate of hospital admission (Table 2).

**Table 1:** Characteristics of included studies

Study/year	Country	Study design	Sample size	Age (years)	Male (%)	BMI (kg/m^2^)	mMRC	FEV1, % predicted	*Pseudomonas aeruginosa* (%)	Number of affected lobes	Exacerbations (previous year)	Follow-up time (years)	Mortality (%)	Hospitalization (%)	Scales
Martínez-García/2014a	Spain	R	397	59.20 ± 17.40	44.30	26.10 ± 4.28	1.57 ± 1.17	68.50 ± 25.60	31.80	2.45 ± 1.12	2.47 ± 2.10	5	19.90	NA	FACED
Martínez-García/2014b	Spain	R	422	58.30 ± 17.70	42.60	25.35 ± 4.98	1.50 ± 1.15	68.70 (26.30)	31.80	2.59 ± 1.17	2.57 ± 2.30	5	17.80	NA	FACED
Chalmers/2014	U.K.	P	608	67 (58–75)	39.97	NA	2 (1, 3)	72.60 ± 25.00	11.51	3.0 ± 1.6	1.7 ± 2.0	4	10.20	0.08	BSI
Ellis/2016	U.K.	R	74	52.50 ± 12.40	NA	23.40 ± 3.90	2.10 ± 0.90	68.80 ± 27.70	22.00	3.40 ± 1.50	4.40 ± 4.40	18.8	35	NA	BS FACEDI
McDonnell/2016a	Dundee, U.K.	P	494	65.30 ± 12.90	39.30	25.90 ± 5.20	2.30 ± 1.10	71.60 ± 24.7	12.80	4.40 ± 3.00	2.10 ± 2.60	4	8.5	0.05	BSI FACED
McDonnell/2016b	Newcastle, England	P	126	59.10 ± 14.50	40.5	26.20 ± 5.10	2.50 ± 1.10	64.00 ± 26.90	10.30	2.80 ± 1.40	3.40 ± 1.70	4	12.7	0.11	BSI FACED
McDonnell/2016c	Belgium	P	190	66.40 ± 16.00	49	23.90 ± 4.30	2.30 ± 1.20	69.30 ± 25.30	8.40	4.50 ± 1.30	1.90 ± 2.10	5	23.16	0.06	BSI FACED
McDonnell/2016d	Monza, Italy	P	250	65.10 ± 12.20	41.2	23.70 ± 4.40	2.00 ± 1.30	79.20 ± 27.50	21.60	5.50 ± 2.70	1.90 ± 2.00	4	5.60	0.09	BSI FACED
McDonnell/2016e	Galway, Ireland	P	280	60.50 ± 14.60	32.9	27.10 ± 5.60	2.00 ± 1.00	80.30 ± 25.90	13.90	3.40 ± 3.00	2.90 ± 1.30	5	15.71	0.03	BSI FACED
McDonnell/2016f	Athens, Greece	P	159	59.30 ± 16.20	36	24.60 ± 3.40	2.40 ± 1.50	70.10 ± 24.90	36.50	4.80 ± 2.50	2.40 ± 1.50	5	5.66	0.04	BSI FACED
McDonnell/2016g	Vojvodina, Serbia	P	113	62.00 ± 13.00	29.2	25.10 ± 4.90	2.50 ± 1.40	64.80 ± 26.20	1.00	4.70 ± 2.40	1.00 ± 1.25	5	17.70	0.02	BSI FACED
Athanazio/2017	Latin America	R	651	48.20 ± 16.00	32.9	22.40 ± 11.50	1.52 ± 1.00	54.70 ± 22.10	39.80	3.30 ± 1.50	1.12 ± 1.40	5	14.60	0.30	FACED
Sim/2017	Singapore	P	96	70 (59.3–77)	37.5	19.20 (15.70–23.10)	NA	47.00 (37.00–63.30)	NA	NA	NA	5	42.70	NA	BSI FACED
Wang/2018	China	R	596	54.81 ± 13.71	56.88	NA	NA	NA	NA	NA	NA	5	7.05	0.06	BSI FACED
Rosales-Mayor/2017	Spain	P	182	68.00 ± 14.60	40.10	25.60 ± 4.60	1.30 ± 1.10	70.30 ± 21.80	20.90	3.20 ± 1.60	1.80 ± 1.80	1	NA	0.27	BSI FACED
Costa/2018	Portugal	R	40	65.90 ± 14.10	45.00	26.20 ± 5.60	2	63.40 ± 22.10	12.50	3.60 ± 1.40	1.20 ± 1.50	NA	NA	NA	BSI FACED
Minov/2015	Macedonia	R	37	63.40 ± 8.10	80.00	24.30 ± 3.70	1.83 ± 0.63	57.60 ± 8.70	8.10	2. 25 ± 0.78	2.12 ± 0.54	NA	NA	NA	BSI FACED

Data are presented as mean ± SD or median (interquartile range). Abbreviations: NA, not available; P, prospective; R, retrospective.

a, b, c etc means different cohorts in one study.

**Table 2:** The distribution of bronchiectasis patients, number of all-cause and respiratory-cause deaths, and number of hospital admissions in different severity groups stratified by FACED and/or BSI

Study/year	Country	Scales	Mild				Moderate				Severe			
			Total	All-cause mortality	Respiratory-cause mortality	Hospitalizations	Total	All-cause mortality	Respiratory-cause mortality	Hospitalizations	Total	All-cause mortality	Respiratory-cause mortality	Hospitalizations
Martínez-García/2014a	Spain	FACED	234	10	2	NA	99	25	15	NA	64	44	33	NA
Miguel/2014b	Spain	FACED	249	14	6	NA	105	23	14	NA	68	38	31	NA
Chalmers/2014	U.K.	BSI	191	4	NA	13	224	13	NA	31	193	44	NA	145
Ellis/2016	U.K.	FACED	49	8	NA	NA	19	13	NA	NA	6	5	NA	NA
		BSI	19	4	NA	NA	32	9	NA	NA	23	13	NA	NA
McDonnell/2016a	Dundee, U.K.	BSI	136	1	NA	3	211	13	NA	24	147	28	NA	75
		FACED	303	12	NA	44	145	15	NA	45	46	15	NA	13
McDonnell/2016b	Newcastle, England	BSI	21	0	NA	0	25	1	NA	5	80	15	NA	52
		FACED	91	6	NA	37	27	7	NA	15	8	3	NA	5
McDonnell/2016c	Leuven, Belgium	BSI	51	2	NA	6	63	16	NA	18	76	26	NA	34
		FACED	100	9	NA	23	65	19	NA	22	25	16	NA	13
McDonnell/2016d	Monza, Italy	BSI	67	0	NA	10	104	3	NA	27	79	11	NA	55
		FACED	135	3	NA	40	88	4	NA	34	27	7	NA	18
McDonnell/2016e	Galway, Ireland	BSI	109	8	NA	2	92	11	NA	6	79	25	NA	30
		FACED	217	23	NA	18	53	19	NA	15	10	2	NA	5
McDonnell/2016f	Athens, Greece	BSI	36	0	NA	1	43	0	NA	7	80	9	NA	27
		FACED	104	0	NA	17	35	4	NA	9	20	5	NA	9
McDonnell/2016g	Vojvodina, Serbia	BSI	41	0	NA	0	48	12	NA	8	24	8	NA	12
		FACED	60	4	NA	2	44	13	NA	3	9	3	NA	1
Athanazio/2017	Latin America	FACED	350	13	7	4	231	48	29	33	70	34	27	19
Sim/2017	Singapore	BSI	9	2	NA	NA	19	3	NA	NA	68	36	NA	NA
		FACED	35	11	NA	NA	40	21	NA	NA	21	9	NA	NA
Wang/2018	China	BSI	46	1	NA	15	244	5	NA	57	306	36	NA	105
		FACED	441	17	NA	123	136	19	NA	48	19	6	NA	6
Rosales-Mayor/2017	Spain	BSI	36	NA	NA	NA	47	NA	NA	NA	99	NA	NA	NA
		FACED	108	NA	NA	NA	61	NA	NA	NA	13	NA	NA	NA
Costa/2018	Portugal	BSI	13	NA	NA	NA	13	NA	NA	NA	14	NA	NA	NA
		FACED	20	NA	NA	NA	15	NA	NA	NA	5	NA	NA	NA
Minov/2015	Macedonia	BSI	16	NA	NA	NA	14	NA	NA	NA	7	NA	NA	NA
		FACED	17	NA	NA	NA	14	NA	NA	NA	6	NA	NA	NA

Abbreviation: NA, not available.

a, b, c etc means different cohorts in one study.

### Mortality prediction

**Figure 3:**
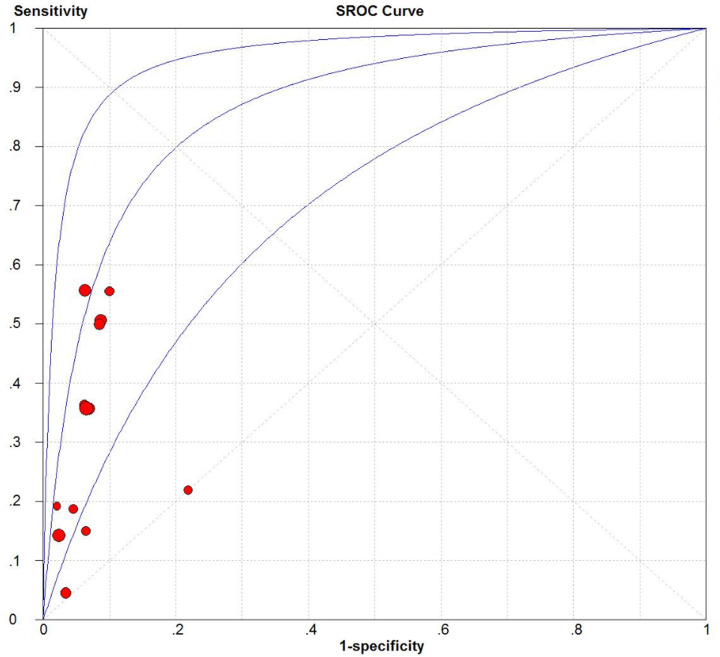
SROC curve of the FACED score for predicting all-cause mortality

**Figure 4:**
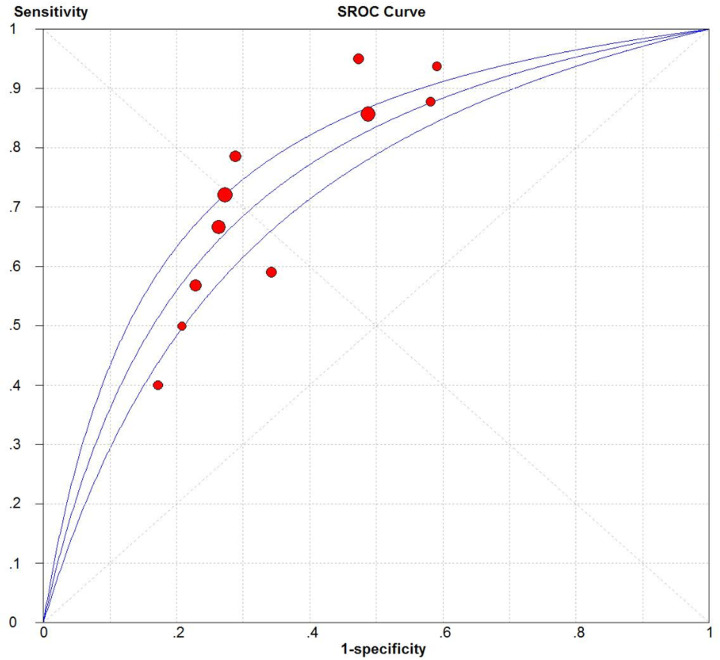
SROC curve of the BSI score for predicting all-cause mortality

We evaluated the predictive accuracy regarding all-cause mortality of the FACED system across 13 cohorts (*n*=3848) [8,10,11,16,17,20], and the corresponding predictive accuracy of the BSI system across 11 cohorts (*n*=2986) [9,11,16,17,20]. Pooled summary estimates, including sensitivity, specificity, PLR, NLR, DOR, and AUC, were calculated using FACED and BSI scores at various cut-off values (Table 3). The FACED score at a cut-off value ≥ 5 had good predictive accuracy based on the pooled sensitivity (0.34, 95% confidence interval [CI] = 0.3–0.38), specificity (0.94, 95% CI = 0.93–0.95), PLR (4.76, 95% CI = 3.48–6.51), NLR (0.74, 95% CI = 0.62–0.88), and DOR (6.67, 95% CI = 4.25–10.45). The BSI score at a cut-off value ≥ 9 had good predictive accuracy based on the pooled sensitivity (0.70, 95% CI = 0.65–0.75), specificity (0.66, 95% CI = 0.64–0.67), PLR (2.05, 95% CI = 1.78–2.37), NLR (0.48, 95% CI = 0.38–0.61), and DOR (5.01, 95% CI = 3.85–6.53). Based on the AUC values, we found that the FACED score was better at predicting all-cause mortality than the BSI score (0.87 vs 0.75; Figures 3 and 4).

**Table 3:** Summary accuracy of FACED score and BSI for predicting mortality and hospitalizations at each cut-off value

Outcomes	Scale	Study/participants	Sensitivity (95% CI), *I^2^*	Specificity (95% CI), *I^2^*	PLR (95% CI), *I^2^*	NLR (95% CI), *I^2^*	DOR (95% CI), *I^2^*	AUC
**All-cause mortality**	**FACED**							
	**≥3**	13/3848	0.76 (0.72–0.80), 72.7%	0.68 (0.66– 0.69), 88.8%	2.31 (2.02– 2.63), 67.5%	0.38 (0.29– 0.49), 66.6%	6.54 (4.71– 9.07), 47.7%	0.77
	**≥5**	13/3848	0.34 (0.30–0.38), 83.0%	0.94 (0.93– 0.95), 74.9%	4.76 (3.48– 6.51), 58.3%	0.74 (0.62– 0.88), 91.7%	6.67 (4.25–10.45), 64.4%	0.87
	**BSI**							
	**≥5**	11/2986	0.94 (0.91– 0.96), 51.8%	0.27 (0.25–0.29), 94.9%	1.31 (1.18– 1.45), 88.6%	0.29 (0.19– 0.42), 0.0%	5.10 (3.26– 7.98), 0.0%	0.66
	**≥9**	11/2986	0.70 (0.65–0.75), 77.2%	0.66 (0.64–0.67), 93.8%	2.05 (1.78– 2.37), 66.6%	0.48 (0.38– 0.61), 48.1%	5.01 (3.85– 6.53), 0.0%	0.75
**Respiratory-cause mortality**	**FACED**							
	**≥3**	3/1470	0.91 (0.85– 0.95), 24.4%	0.63 (0.60– 0.65), 76.7%	2.50 (2.07– 3.01), 77.4%	0.16 (0.09– 0.27), 20.2%	16.36 (7.97– 33.58), 37.2%	0.64
	**≥5**	3/1470	0.56 (0.48– 0.63), 71.1%	0.92 (0.90– 0.93), 8.3%	6.42 (5.13– 8.04), 0.0%	0.48 (0.34– 0.67), 70.4%	13.38 (8.80– 20.34), 21.6%	0.93
**Hospitalizations**	**FACED**							
	**≥3**	9/2859	0.50 (0.46– 0.54), 90.9%	0.67 (0.65– 0.69), 90.5%	1.70 (1.38– 2.10), 78.6%	0.67 (0.54– 0.84), 79.4%	2.71 (1.79– 4.09), 71.6%	0.69
	**≥5**	9/2859	0.14 (0.12– 0.17), 83.0%	0.94 (0.92– 0.95), 72.9%	2.60 (1.84– 3.69), 37.7%	0.89 (0.82– 0.97), 82.3%	2.95 (1.93– 4.49), 41.5%	0.82
	**BSI**							
	**≥5**	9/2816	0.94 (0.92– 0.95), 57.4%	0.32 (0.30– 0.34), 95.9%	1.43 (1.21– 1.69), 95.4%	0.20 (0.09– 0.41), 80.4%	7.61 (3.16 –18.32), 83.2%	0.80
	**≥ 9**	9/2816	0.70 (0.66– 0.73), 80.1%	0.74 (0.72– 0.76), 96.2%	2.93 (1.90– 4.51), 95.3%	0.39 (0.28– 0.55), 87.9%	7.85 (3.60– 17.08), 93.1%	0.80

Meta-regression and subgroup analyses of the cohorts were performed based on study design, age, follow-up time, and mortality. Regarding the FACED score with a cut-off value ≥ 3, study design and age were identified as sources of heterogeneity. In the subgroup analysis, heterogeneity decreased significantly when the analyses were restricted to older patients, especially those ≥ 65 years (*I^2^* = 0, AUC = 0.72). However, significant heterogeneity associated with study design persisted. Regarding the BSI score with a cut-off value ≥ 5, study design had a significant influence on heterogeneity: a prospective design was associated with slightly lower heterogeneity (*I^2^* = 84.54%). The results of the corresponding subgroup analysis are provided in Supplementary Table S1.

The results of Deeks’ test showed no significant publication bias across the included studies, based on FACED (*P*=0.531 for a cut-off value ≥ 3; *P*=0.315 for a cut-off value ≥ 5) or BSI (*P*=0.871 for a cut-off value ≥ 5; *P*=0.375 for a cut-off value ≥ 9).

Respiratory-related mortality was evaluated using data from three cohorts (*n*=1470) that used the FACED system [8,10]. We calculated an AUC of 0.93 for respiratory-related mortality prediction using the FACED score at a cut-off value ≥ 5.

### Hospitalization prediction

**Figure 5:**
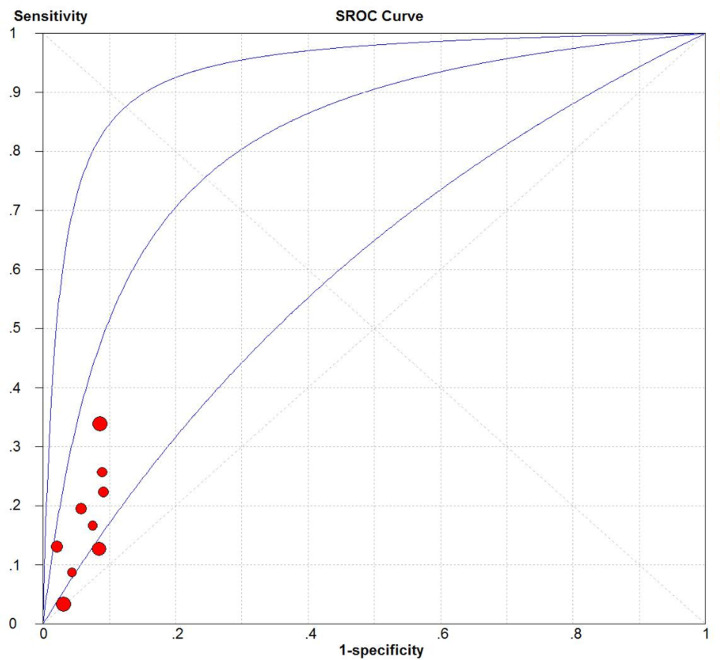
SROC curve of the FACED score for predicting hospitalization

The accuracy of the two systems for predicting hospitalization of patients with bronchiectasis was evaluated using data from nine cohorts (*n*=2859) in the case of FACED [10,16,17] and nine cohorts (*n*=2859) in the case of BSI [9,16,17] (Table 3). AUC values indicated that FACED scores at a cut-off value ≥ 5 could predict hospitalization (AUC = 0.82; Figure 5), as could BSI scores at cut-off values ≥ 5 or ≥ 9 (AUC = 0.80 for both cut-off values).

Meta-regression and subgroup analyses were not performed due to the limited number of included cohorts. Deeks’ test showed no significant publication bias (*P*=0.497 for a cut-off value ≥ 3; *P*=0.129 for a cut-off value ≥ 5) or BSI (*P*=0.153 for a cut-off value ≥ 5; *P*=0.896 for a cut-off value ≥ 9).

### Agreement between FACED and BSI scores

By analyzing the paired FACED and BSI data available for 333 bronchiectasis patients (Table 4), we found that the FACED and BSI systems stratified 155 patients (46.54%) into the same group (κ = 0.25, *P*<0.001). However, the FACED score assigned 194 patients (58.26%) to the mild group, compared to the 84 (25.22%) classified as mild based on the BSI score (*P*<0.001). Additionally, the BSI score classified nearly five times more patients as severe (143 [42.94%] vs 30 [9%], *P*<0.001). In contrast, 58 of the 333 (17.42%) bronchiectasis patients stratified into the mild group based on the FACED score were stratified into the severe group based on the BSI score.

**Table 4:** Agreements analysis between FACED score and BSI

BSI	FACED			Total
	Mild	Moderate	Severe	
Mild	81	2	1	84
Moderate	55	48	3	106
Severe	58	59	26	143
Total	194	109	30	333

## Discussion

Early identification of bronchiectasis patients with poor prognosis, leading to their close monitoring and intensive treatment, can enhance the efficiency of clinical practice, improve resource allocation, and help to optimize therapeutic outcomes. This meta-analysis summarized the prognostic performance of the FACED and BSI systems in patients with bronchiectasis for the first time. Our results show that, when appropriate cut-offs are used, the FACED score can play an important role in predicting all-cause mortality, while both the FACED and BSI scores can predict hospitalization in patients with bronchiectasis. Further research is essential to gain a better understanding of the potential prognostic roles of FACED and BSI scores in bronchiectasis.

The multidimensional and heterogeneous nature of bronchiectasis makes predicting prognoses challenging [6]. For example, risk factors associated with mortality in bronchiectasis patients include age, sex, body mass index, smoking habits, Medical Research Council dyspnea score, radiographic extent, bacterial colonization, spirometric parameters, and comorbidities (restrictive and obstructive diseases) [21–24]. FACED takes into account five of these risk factors, while BSI takes into account the same five plus two more. Therefore, both systems may be useful for the prediction of bronchiectasis outcomes and stratification by severity. However, different studies have reported different results [10,11], highlighting the need for accurate comparisons of the two systems based on current available evidence.

The accuracy of FACED and BSI scores depends on the cut-off values used. The FACED score seems to predict all-cause mortality more accurately than the BSI score. This, coupled with its simplicity, may make FACED particularly powerful. It can be used to identify patients who do not need intensive therapy. However, it can also delay needed treatment if a patient is incorrectly classified as low risk. We found that the predictive performance of the BSI score regarding all-cause mortality was inadequate. Further research may lead to an improved system being developed.

Based on our systematic review, only the FACED score has been used in research studies to predict respiratory-related mortality, for which it showed an excellent prognostic performance at a cut-off value ≥ 5 (AUC = 0.93). Thus, it can be used for the reliable identification of high-risk bronchiectasis patients, although it may incorrectly classify low-risk bronchiectasis patients as having severe disease, leading to unnecessary treatment. Therefore, it should be used with caution when predicting respiratory-related mortality.

In addition to mortality, a substantial proportion of patients with bronchiectasis experience exacerbation in terms of frequency and severity [25], leading to hospitalization in severe cases. This hospitalization is associated with rapidly growing healthcare costs [26,27]. Accurate prediction of hospitalization may help clinicians and patients to weigh the potential benefits and costs of treatment more accurately. The results of our meta-analysis indicate that both FACED and BSI scores are useful for predicting hospitalization due to bronchiectasis. However, as the FACED system does not account for previous instances of exacerbation, which is a valuable predictor of future exacerbation [28], it should be used with caution to predict hospitalization, and it may require further improvement. Indeed, the EFACED system, which accounts for exacerbations, may predict hospitalization better, and it is recommended by the Spanish guidelines [7,29]. Future research should compare EFACED and BSI in terms of hospitalization prediction.

Regardless of the cut-off values tested, neither FACED nor BSI achieved ‘perfect’ prediction, defined as PLR > 10 and NLR < 0.1 [30], regarding predicting all-cause mortality or hospitalization. This highlights the need for improvement. Bronchiectasis is associated with various etiologies and comorbidities that influence disease outcomes. The risk of death, exacerbation, and hospitalization is significantly higher in bronchiectasis patients with comorbidities than in those without [30,31]. Understanding the underlying etiologies and comorbidities may allow more comprehensive evaluation, leading to personalized treatment and better prediction of prognosis. However, neither FACED nor BSI takes comorbidities into consideration. To address this problem, the Bronchiectasis Aetiology Comorbidity Index (BACI) was developed to account for 13 comorbidities associated with high risk [32]. Future research should explore whether adding comorbidities to the FACED and BSI systems improves their performance.

*Pseudomonas aeruginosa* is associated with bronchiectasis and poor clinical outcomes [33]. Chronic colonization by *P. aeruginosa* is scored with 1 point in the FACED system and 3 points in the BSI system. *P. aeruginosa* has been reported to be significantly more abundant in patients with moderate or severe bronchiectasis, based on FACED scores [34]. Nevertheless, a large multinational study found that *P. aeruginosa* infection had no independent impact on mortality, and instead suggested that the association between *P. aeruginosa* and high mortality risk depends on exacerbation of the disease [35]. We hypothesize that reducing the points assigned to *P. aeruginosa* colonization may improve the ability of the BSI system to predict mortality.

Our conclusions should be interpreted carefully in light of the limitations of our systematic review and meta-analysis. Only 10 studies were included even after a comprehensive literature search, and some studies contained overlapping data. The evidence base comes primarily from Europe and to a lesser degree from Asia, yet the disease prevalence and hospital treatment and management practices differ by geographic region and healthcare setting. Therefore, the performance of FACED and BSI scores should be assessed in a greater diversity of settings. We excluded studies not published in English or Chinese, which may have led to bias. Additionally, the studies in our review did not adjust for the fact that during follow-up, patients may have received treatments that influenced disease outcomes.

Beyond these research limitations, the intrinsic limitations of the FACED and BSI systems should be taken into account when using them to stratify bronchiectasis patients. The prevalence of bronchiectasis and health resources in different countries should be considered when interpreting and applying the results of the FACED and BSI systems in clinical settings. For instance, the FACED score is easy to calculate owing to its simplicity, while an online calculator may be needed to determine the BSI score, and the BACI score is more complicated to calculate. Neither the FACED nor the BSI system includes all relevant factors, such as biological activity or impact of bronchiectasis on the patient's quality of life. Using a “clinical fingerprint” and “control model” approach may improve clinicians’ ability to take into account the complexity and heterogeneity of bronchiectasis, ultimately improving the quality of patient care [36].

## Conclusions

The available evidence suggests that for patients with bronchiectasis, the FACED score can play an important role in predicting mortality, while both the FACED and BSI scores may be useful for predicting hospitalization. Further studies in diverse populations and healthcare settings are needed to validate our findings.

## Supplementary Material

Supplementary Table S1 and Supplementary material (e-Appendix 1)

## Data Availability

The datasets supporting the conclusions of this article are included within the article and in the supplementary files.

## Abbreviations

AUC: area under the curve
BACI: Bronchiectasis Aetiology Comorbidity Index
BSI: Bronchiectasis Severity Index
CI: confidence interval
DOR: diagnostic odds ratio
EFACED: exacerbation added to FACED
FACED: forced expiratory volume in 1 s, Age, Chronic colonization, Extension, and Dyspnea
NLR: negative likelihood ratio
PLR: positive likelihood ratio
QUADAS-2: Quality Assessment of Diagnostic Accuracy Studies-2
SROC: summary receiver operating characteristic
