# Supplementary material for: Prognostic performance of the FACED score and bronchiectasis severity index in bronchiectasis: a systematic review and meta-analysis
Source: Biosci Rep. 2020 Oct 30;40(10):BSR20194514. doi: 10.1042/BSR20194514 (PMC7601347; doi:10.1042/BSR20194514)
Supplement: Supplementary Table S1 and Supplementary material (e-Appendix 1) [file BSR-2019-4514_supp.pdf]

Supplementary Table 1. The subgroup analysis for pooled estimates of FACED score and BSI for predicting mortality at each cut-off value

Outcomes / Scales	Study / Participants	Sensitivity (95% CI)	Specificity (95% CI)	PLR (95% CI)	NLR (95% CI)	DOR (95% CI)	I^2
**All-cause Mortality**							
FACED							
≥ 3	13 /3848	0.76 (0.69 - 0.82)	0.68 (0.63 - 0.73)	2.40 (2.09 - 2.75)	0.35 (0.28 - 0.45)	6.53 (5.03 - 9.27)	96.12
Design - Retrospective	5/2140	0.79 (0.68 - 0.86)	0.71 (0.64 - 0.76)	2.68 (2.34 - 3.08)	0.31 (0.21 - 0.44)	8.81 (6.11 - 12.71)	90.95
Design - Prospective	8/1708	0.72 (0.62 - 0.79)	0.66 (0.58 - 0.73)	2.09 (1.76 - 2.48)	0.43 (0.34 - 0.55)	4.83 (3.48 - 6.69)	93.57
Age - ≤ 65-year-old	9/2818	0.76 (0.65 - 0.84)	0.72 (0.67 - 0.77)	2.70 (2.42 - 3.01)	0.34 (0.24 - 0.47)	8.00 (5.71 - 11.20)	96.39
Age - ≥ 65-year-old	4/1030	0.75 (0.67 - 0.82)	0.59 (0.52 - 0.65)	1.82 (1.51 - 2.18)	0.42 (0.31 - 0.58)	4.28 (2.68 - 6.86)	0
BSI							
≥ 5	11/2986	0.95 (0.91 - 0.98)	0.27 (0.20 - 0.35)	1.30 (1.18 - 1.42)	0.19 (0.10 - 0.34)	7.01 (3.67 - 13.36)	91.67
Design - Retrospective	2/670	NA	NA	NA	NA	NA	
Design - Prospective	9/2316	0.96 (0.91- 0.98)	0.30 (0.25 - 0.36)	1.37 (1.28 - 1.47)	0.14 (0.06 - 0.29)	10.15 (4.61 - 22.35)	84.54

Notes: Abbreviations: PLR, Positive Likelihood Ratio; NLR, Negative Likelihood Ratio; DOR, Diagnostic Odds Ratio; NA, Not Available.

e-Appendix 1

Methods

Search strategy

Database: PubMed

1. "bronchiectasis"[MeSH Terms] OR “bronchiectasis” [tiab] OR “non-CF bronchiectasis” [tiab] OR “NCFB” [tiab] OR “bronchiect*”
2. “Bronchiectasis screen*” OR “Bronchiectasis score*” OR “Bronchiectasis scale*” OR “Bronchiectasis assessment*” OR “Bronchiectasis index*” OR “FACED” OR “Bronchiectasis Severity Index” OR “BSI”
3. 1 AND 2

Database: EMBASE

1. exp Bronchiectasis/
2. bronchiect$.mp.
3. bronchoect$.mp.
4. kartagener$.mp.
5. (bronchial$ adj3 dilat$).mp.
6. or/1-5
7. (assessment test or Bronchiectasis screen* or Bronchiectasis score* or Bronchiectasis scale* or Bronchiectasis assessment* or Bronchiectasis index or FACED or Bronchiectasis Severity Index or BSI).mp.
8. 6 AND 7

Cochrane Database of Systematic Reviews

1. bronchiectasis OR non-CF bronchiectasis OR NCFB OR bronchiect*

2. assessment test or Bronchiectasis screen* or Bronchiectasis score* or Bronchiectasis scale* or Bronchiectasis assessment* or Bronchiectasis index or FACED or Bronchiectasis Severity Index or BSI

3. 1 AND 2
